# Fouling Mitigation by Cationic Polymer Addition into a Pilot-Scale Anaerobic Membrane Bioreactor Fed with Blackwater

**DOI:** 10.3390/polym12102383

**Published:** 2020-10-16

**Authors:** Magela Odriozola, Nicolás Morales, Jose R. Vázquez-Padín, Maria Lousada-Ferreira, Henri Spanjers, Jules B. van Lier

**Affiliations:** 1 Department of Water Management, Delft University of Technology, Stevinweg 1, 2628 CN Delft, The Netherlands; M.LousadaFerreira@tudelft.nl (M.L.-F.); H.L.F.M.Spanjers@tudelft.nl (H.S.); J.B.vanLier@tudelft.nl (J.B.v.L.); 2 Aqualia, Rúa das Pontes 4, 36350 Nigrán, Pontevedra, Spain; nicolas.morales.pereira@fcc.es (N.M.); jvazquezp@fcc.es (J.R.V.-P.)

**Keywords:** anaerobic membrane bioreactor (AnMBR), cationic polymer, flux enhancer, membrane fouling, pilot plant, sludge filterability

## Abstract

Cationic polymers have proven to be suitable flux enhancers (FEs) in large-scale aerobic membrane bioreactors (MBRs), whereas in anaerobic membrane bioreactors (AnMBRs) research is scarce, and so far, only done at lab-scale. Results from MBRs cannot be directly translated to AnMBRs because the extent and nature of membrane fouling under anaerobic and aerobic conditions are different. Our research focused on the long-term effect of dosing the cationic polymer Adifloc KD451 to a pilot AnMBR, fed with source-separated domestic blackwater. A single dosage of Adifloc KD451 at 50 mg L^−1^ significantly enhanced the filtration performance in the AnMBR, revealed by a decrease in both fouling rate and total filtration resistance. Nevertheless, FE addition had an immediate negative effect on the specific methanogenic activity (SMA), but this was a reversible process that had no adverse effect on permeate quality or chemical oxygen demand (COD) removal in the AnMBR. Moreover, the FE had a long-term positive effect on AnMBR filtration performance and sludge filterability. These findings indicate that dosing Adifloc KD451 is a suitable strategy for fouling mitigation in AnMBRs because it led to a long-term improvement in filtration performance, while having no significant adverse effects on permeate quality or COD removal.

## 1. Introduction

The anaerobic membrane bioreactor (AnMBR) is regarded as a technology of interest for wastewater treatment, allowing the production of reclaimed water at a reduced level of energy consumption while recovering resources. AnMBR couples the advantages of anaerobic digestion, such as low sludge production, no aeration requirement and biogas production, with the benefits of membrane technology, that is, complete solids removal and a high degree of removal of pathogenic organisms [1]. However, fouling remains the major operational challenge in AnMBRs, because it is responsible for lower transmembrane flux (J) and higher transmembrane pressure (TMP), and the need for intensive biogas sparging, an increased frequency of membrane cleaning and membrane replacement [2], and thus increasing energy and operational costs.

The factors impacting membrane fouling have been widely studied. The literature has shown that fouling is sensitive to sludge characteristics, membrane operation and membrane properties [3,4,5,6]. For the last two decades, extensive research has been done on the application of flux enhancers (FE), including adsorbents, coagulants and flocculants, to improve sludge filtration properties and mitigate fouling by modifying sludge characteristics.

One of the most comprehensive studies was performed by Iversen, Koseoglu and collaborators [7,8,9]. The research started with batch-test experiments using 30 different FE and culminated in the application of 3 of them into a pilot-scale aerobic membrane bioreactor (MBR)—the starch Mylbond168, and the synthetic cationic polymers MPE50 and Adifloc KD452. The cationic polymers improved filtration performance in the MBR, whereas starch had a detrimental effect. Nevertheless, in short-term lab experiments performed with a cross-flow filtration test cell, the filtration performance was improved with the three chemicals, and the improvement obtained with the cationic polymers was higher than in subsequent studies in the pilot. The authors postulated that the different results in pilot and lab-scale experiments might have been due to the hydrodynamic differences between the installations (MBR and test cell). Moreover, the authors emphasized the need for more research on FE addition in long-term and large-scale trials (i.e., pilot and full scale), which was also addressed by other authors [2,10]. Table S1 summarizes the published applications of FE in large-scale membrane bioreactors, namely Dong et al. [11], Dong et al. [12], Teli et al. [13], Iversen et al. [9], Alkmim et al. [14], Collins et al. [15], Wozniak [16], Yoon and Collins [17], Munz et al. [18] and Remy [19]. MPE50 was the most commonly employed FE, mostly used by researchers from Nalco, the supplier of the polymer [15,16,17]. In all the studies that applied cationic polymers, namely MPE50 and KD452, membrane filtration was improved, which was shown by a TMP decrease or flux increase. Therefore, it can be concluded that cationic polymers are suitable FE for fouling mitigation. However, all those studies were performed in (aerobic) MBRs. The extent and nature of the membrane fouling mechanisms in MBRs and AnMBRs can be very different because of the very different biomass developments and the different natures of the potential foulants under aerobic and anaerobic conditions [3,20]. Particularly, under anaerobic conditions, higher concentrations of colloidal organic matter are reported compared to aerobic conditions, which might result in higher fouling rates [3]. Therefore, the results from aerobic MBRs cannot be directly translated to AnMBRs, and it is important to study the feasibility of using cationic polymers for fouling reduction in AnMBRs.

To the authors’ best knowledge, to date there are only two available publications about the application of cationic polymers in AnMBRs, both performed at lab scale-Diaz et al. [21] achieved a flux increase by dosing 1.5 g L^−1^ of MPE50 to a 4.5 L AnMBR fed with synthetic wastewater, and Kooijman et al. [22] obtained a decrease in the specific resistance to filtration (SRF) by applying 10 g kg^−1^ of the cationic polymer Calfloc P1502, combined with 40% FeCl_3_, to an anaerobic dynamic membrane bioreactor (AnDMBR) fed with waste activated sludge. Furthermore, cationic polymers have been studied in batch tests with anaerobic sludge samples, leading to improved filtration characteristics in dead-end filtration tests [21,22,23] and cross-flow filtration tests [21,24,25]. Therefore, cationic polymers are considered suitable FEs for fouling mitigation in lab-scale AnMBRs and large-scale MBRs (Table S1). However, these FEs should be further studied in long-term large scale AnMBRs.

The most common strategy of dosing FE to membrane bioreactors, as presented in Table S1, is to try and sustain a desired concentration of FE inside the reactor based on different assumptions. We introduce the term ‘feedforward dosing’ to describe this strategy, which was previously referred to as preventive FE use [14]. Feedforward dosing has been applied by performing an initial pulse of FE to achieve the desired concentration, followed by periodic additions to compensate for the loss of FE due to sludge withdrawal and possible FE biodegradation. The desired concentrations have been estimated in batch tests with sludge samples from the reactor before FE addition, or based on reported values. FE biodegradations have been considered negligible or based on supplier’s recommendation; for example, Nalco suggested that 1% of the MPE50 is biodegraded daily [14]. Furthermore, all researchers that performed feedforward dosing in Table S1 did not explicitly consider the possible FE loss in the permeate and used a unique target FE dosage which did not change over the reactor’s operation. Moreover, feedforward dosing does not consider possible disturbances, such as fluctuations in the influent characteristics, which can be present in full-scale plants even when operating at the design conditions. Therefore, despite being the most used dosing strategy, feedforward dosing is based on assumptions that might lead to under or overdose of FE. Overdosing FE can have detrimental effects on filtration performance, permeate quality and biological activity [24], whereas underdosing FE may result in the insufficient improvement of the sludge filtration performance.

An alternative FE dosing strategy is a feedback control loop, whereby the addition of FE is adjusted based on an input variable that quantifies the sludge filtration characteristics, such as filterability. We introduce the term ‘feedback dosing’ for this strategy, which was previously referred to as corrective FE use [14]. In feedback dosing, a pulse of FE is applied to the reactor only when the sludge filterability is deteriorated. Feedback dosing does not require the assumptions made in feedforward dosing and it can cope with possible disturbances of the sludge filtration characteristics, and thus avoid under or overdosing of FE. Nevertheless, the major challenge in feedback dosing is to identify an appropriate variable to quantify sludge filtration characteristics that could be measured regularly, preferably in-situ and online. Various researchers suggested the possible application of the online measurement of sludge filtration characteristics for automatic FE dosing control in membrane bioreactors [8,26]. However, this has not been further studied or tested.

The aim of this research is to analyze the long-term effect of dosing the cationic polymer Adifloc KD451 to a pilot AnMBR fed with source-separated domestic blackwater. We researched the effects on permeate quality, sludge characteristics, biological activity (i.e., COD removal and specific methanogenic activity, SMA) and AnMBR filtration performance (i.e., fouling rate and filtration resistance). Additionally, we determined the applicability of in-situ measurements of sludge filterability as an input variable in an FE feedback dosing control strategy.

## 2. Materials and Methods

### 2.1. Pilot AnMBR Plant Description

The pilot AnMBR plant was located at the Business Center Porto do Molle, Nigrán, Pontevedra, Spain. The reactor was fed with blackwater collected in segregated pipes in the main office building, where approximately 200 persons worked. The toilets in the building were conventional gravity flush toilets (3.0–4.5 L of water per flush).

**Figure 1:**
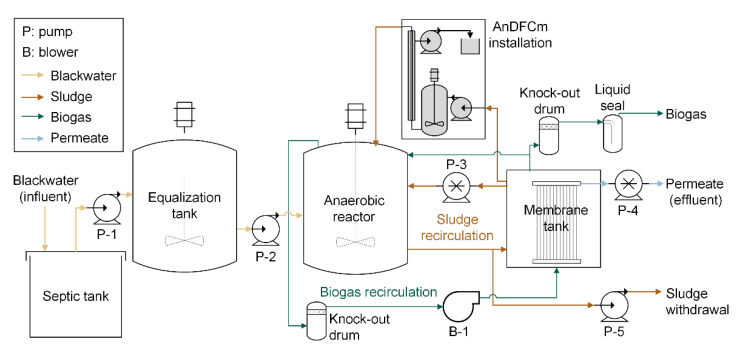
Scheme of the pilot AnMBR plant including the AnDFCm installation connected in bypass for in-situ sludge filterability measurements.

Figure 1 shows the scheme of the pilot plant, including the AnDFCm installation connected to the AnMBR. The blackwater was stored in a 3–4 m^3^ septic tank followed by a 1 m^3^ equalization tank. The AnMBR was composed of a 2.8 m^3^ anaerobic stirred reactor connected to a 1.0 m^3^ membrane tank. The membrane tank had one submerged ultrafiltration flat-sheet membrane module (Martins System, Berlin, Germany), made of polyethersulfone, with a 6.25 m^2^ surface area and a 35 nm nominal pore size.

The pilot plant was coupled with a supervisory control and data acquisition (SCADA) system and several sensors. The following variables were measured and recorded once per minute by the SCADA: TMP, permeate flow rate, operational phase (i.e., filtration, relaxation or stand-by), accumulated permeate volume, motor frequency of B-1, P-2 and P-4, gas pressure in the head-space in the anaerobic reactor, liquid levels in the equalization tank, anaerobic reactor and membrane tank, and the temperature, pH and redox potential of the sludge in the anaerobic reactor.

The lower detection limit of the biogas discharge flowmeter was usually higher than the flow, and thus the biogas discharge flow could not be detected accurately by the instrument. Moreover, the biogas recirculation flow rate ($Q_{G}$) was not measured online but manually recorded by the operators with a rotameter placed after B-1. These data were used to derive and calibrate an empirical model to calculate the specific gas demand (${\mathrm{SGD}}_{m}$), based on the liquid level in the membrane tank ($H_{\mathrm{MT}}$) and the motor frequency of the blower ($v_{B}$); further details are given in Section 2.2.

Blackwater was homogenized in the equalization tank and pumped into the anaerobic reactor. The sludge was continuously recirculated through the anaerobic reactor and membrane tank, where the permeate was extracted under suction with a peristaltic pump (P-4). The blower and all pumps operated at constant motor input frequencies fixed by the operator. Under normal operational conditions, the membrane presented filtration and relaxation cycles of 300 and 90 s, respectively. The total liquid volume was ~2.8 m^3^ (membrane tank + anaerobic reactor), the hydraulic retention time (HRT) was ~2 days, the periodic sludge wastage was negligible (only sampling) and the reactor operated at room temperature. The head-space biogas was sparged below the membrane module at a $Q_{G}$ of 6–8 Nm^3^ h^−1^, which corresponds with an ${\mathrm{SGD}}_{m}$ of 0.96–1.28 Nm^3^ h^−1^ m^−2^, to provide suitable shear on the membrane surface.

### 2.2. Specific Gas Demand ($SGD_{m}$ ) Model

In addition to the use of FE, research has proven that biogas or air sparging substantially affects fouling [3,27]. Therefore, in our research, it was important to quantify continuously the ${\mathrm{SGD}}_{m}$, to account for the effect of biogas sparging on fouling, both before and after FE addition. ${\mathrm{SGD}}_{m}$ is calculated by dividing $Q_{G}$ by the membrane surface area ($A_{m}$, m^2^). However, as above-mentioned, $Q_{G}$ was not measured online, but manually recorded by the operators with a rotameter placed after the blower B-1.

The experimental ${\mathrm{SGD}}_{m}$, which was calculated with the manually recorded $Q_{G}$, was used to derive and calibrate an empirical model to calculate ${\mathrm{SGD}}_{m}$ continuously, as in Equation (1), with the following online monitored variables: $H_{\mathrm{MT}}$ and $v_{B}$.

(1) $${\mathrm{SGD}}_{m}=\frac{\beta_{0}+\beta_{1}\;H_{\mathrm{MT}}+\beta_{2}\;v_{B}}{A_{m}}$$

where $\beta_{0}$ (Nm^3^ h^−1^), $\beta_{1}$ (Nm^3^ h^−1^ m^−1^), and $\beta_{2}$ (Nm^3^ h^−1^ Hz^−1^) are the model parameters. These parameters were estimated to fit the experimental ${\mathrm{SGD}}_{m}$, using the linear least squares optimization function *lsqlin* in Matlab^®^ R2019b.

### 2.3. Flux Enhancer Dosing

In a previous study (results not shown here, manuscript in preparation), six potential FEs were compared for their effect on the filterability of sludge obtained from a full-scale anaerobic digester at a local sewage treatment plant, including powder-activated carbon (PAC), polyaluminium chloride PAX14, polyaluminium chloride PAX18, and the cationic polymers Adifloc KD352, Adifloc KD451 and MPE50. Optimal dosages were determined as the concentration at which the maximum soluble COD removal was achieved, through jar-test experiments. Afterwards, the sludge filterability in the sludge samples, without FE addition and with FE addition at its optimal dosage, was measured, applying the AnDFCm. Except for PAC, the remaining FEs considerably improved the sludge filterability, with improvements ranging from 72% to 96%. Particularly, Adifloc KD451 improved filterability by 96%, and its optimal dosage was between 1/44 and 1/3 of the optimal dosages for the remaining FEs. Therefore, we selected Adifloc KD451 (Adipap SA, Versailles, France) as the FE for further tests.

A single dose of FE, i.e., pulse-dosage, was added to the AnMBR on day 16. The cationic polymer Adifloc KD451, which has a low molecular weight and high charge density, was used as the FE. A 138.5 g pulse input of Adifloc KD451 was introduced to the bypass line of the AnMBR with an injection time of 45 min. This bypass line was also use for the AnDFCm installation (see Figure 1). The dosed mass was added to achieve a final concentration of Adifloc KD451 in the mixed liquor of 50 mg L^−1^. This concentration was based on previous work [24], and was an intermediate dosage between the optimal dosages for sludge filterability improvement and for csCOD removal of the sludge that was collected from the pilot-scale AnMBR fed with source-separated blackwater before the reactor was spiked with FE.

### 2.4. Monitoring Phases

The AnMBR was inoculated with 500 L of sludge from the mesophilic anaerobic digester of the Guillarei municipal wastewater treatment plant, and was operated for a 5 month acclimation period before Phase I, which is defined below. The AnMBR membrane was chemically cleaned with sodium hypochlorite prior to Phase I, and no further chemical cleanings were performed. On day 123, 0.84 m^3^ of sludge were withdrawn from the AnMBR because of a too-high accumulation of solids, and the removed volume was replaced with blackwater.

The three operational phases, relevant for this work, were defined as follows: Phase I (Period: 0–16 d) is the control phase previous to FE addition; Phase II (Period: 16–123 d) is the period following FE addition and before sludge withdrawal; and Phase III (Period: 123–154 d) is the period after sludge withdrawal.

To study the effect of biogas sparging on filtration performance, we operated the AnMBR with a reduced $Q_{G}$ of 2–4 Nm^3^ h^−1^, that is, an ${\mathrm{SGD}}_{m}$ of 0.32–0.64 Nm^3^ h^−1^ m^−2^, by decreasing the $v_{B}$ over 2 days (Period: 37–39 d).

### 2.5. Analytical Methods

#### 2.5.1. Physicochemical Characterization

Hach Lange test kits were used to measure chemical oxygen demand (COD), ammonium-nitrogen (NH_4_–N), total nitrogen (TN), and total phosphorous (TP). The organic matter was measured as COD in different fractions, as described in Odriozola et al. [24]—total COD (tCOD), supracolloidal COD (scCOD, above 1 µm) and colloidal + soluble COD, named submicron COD (csCOD, below 1 µm).

Total suspended solids (TSS), volatile suspended solids (VSS), fixed suspended solids (FSS), total solids (TS), volatile solids (VS) and fixed solids (FS) were measured following the Standard Methods (APHA, 1999). Alkalinity was measured using potentiometric titration to the end-point pH of 3.7 (APHA, 1999). Particle size distribution (PSD) was measured with a Microtrac Bluewave diffraction analyzer (Malvern Instruments Ltd., UK), and reported as the 50th percentile of the volume-based particle size distribution, or median diameter, D50. We assumed that PSD represents floc size, as explained in Odriozola et al. [24].

Grab samples of sludge, blackwater and permeate were taken from the AnMBR for characterization. suspended solids, csCOD, Alkalinity, pH and PSD were measured in the sludge; tCOD, TP, TN and NH_4_–N in both the blackwater and the permeate, and csCOD, scCOD, Alkalinity, pH and Total Solids in the blackwater. 

Sludge filterability was measured with short-term cross-flow filtration tests, employing the anaerobic Delft filtration characterization method (AnDFCm) installation, connected in bypass to the AnMBR, as shown in Figure 1. During the in-situ filterability measurements, sludge flowed continuously from the membrane tank to the anaerobic reactor, passing through the AnDFCm installation, which contained an X-Flow membrane (Pentair, Enschede, the Netherlands), and had the following characteristics: tubular, 30 nm pore size, 8 mm internal diameter, and 95 cm length. The AnDFCm measured the additional resistance obtained when 20 L of permeate per m^2^ of membrane surface area are produced, denominated as the $∆R_{20}$; the sludge filterability is inversely related to $∆R_{20}$. $∆R_{20}$ was measured by applying a flux of 60 L m^−2^ h^−1^ and a cross-flow velocity of 1.5 m s^−1^. The scheme of the AnDFCm installation in Figure 1 is simplified, and a more detailed representation of the installation and a description of the measuring protocol is presented elsewhere [24].

#### 2.5.2. Specific Methanogenic Activity (SMA)

SMA was measured in Schott glass bottles with 400 mL liquid and 208 mL head-space, under mesophilic conditions using sodium acetate as carbon source and sludge samples from the AnMBR as inoculum. The sludge samples were placed at 4 ℃ before the SMA test, and thus we included a pre-activation period.

For the pre-activation period, all the SMA bottles, including blanks, were filled with 1.0 gCOD L^−1^ of sodium acetate, 2 gVSS L^−1^ of inoculum, 0.6 mL L^−1^ micro and 6 mL L^−1^ macro nutrients solutions [28,29], 10 mM phosphate buffer solution at pH 7.0 [30] and demineralized water. The bottles were flushed with nitrogen gas for 1 min and placed inside an orbital shaker at 130 rpm with temperature set at 35 °C.

For the SMA determination, after all the substrate was converted into methane, a new addition of sodium acetate, to reach 2 gCOD L^−1^, was performed for all the bottles except the blanks. The SMA was calculated from the methane production rate after the second addition of sodium acetate and following the protocol of Spanjers and Vanrolleghem [30]. The methane production rate was measured with an “automated methane potential test system” (AMPTS, Bioprocess Control, Sweden).

To study the adaptability of the biomass to the FE, we measured the SMA for two sludge samples taken from the AnMBR as inoculums: one collected during Phase I immediately before FE addition, on day 16, and the other during Phase II, 3 weeks after FE addition, on day 37. The concentration of sludge (inoculum) in the SMA bottles was 2 gVSS L^−1^, and no extra Adifloc KD45 was added to these bottles. Furthermore, with the sludge collected on day 16, we performed an additional SMA test by pre-mixing the sludge with Adifloc KD451, in 1 L jars at 90 rpm for 30 min, and using the mix as inoculum. In the additional SMA test, the concentrations of sludge and Adifloc KD451 in the SMA bottles were 2 gVSS L^−1^ and 50 mg L^−1^, respectively.

### 2.6. AnMBR Filtration Performance Indices

The AnMBR filtration performance indices were total filtration resistance ($R_{T}$, m^−1^) and fouling rate (FR, Pa s^−1^). $R_{T}$ was calculated with Darcy’s law, as in Equation (2).

(2) $$R_{T}=\frac{TMP}{\eta \;J}$$

where η is the dynamic viscosity of the permeate (Pa s), and J is the transmembrane flux (m s^−1^), which is calculated by dividing the online monitored permeate flow by the membrane surface area. The permeate viscosity was assumed to be equal to pure water viscosity, and was calculated at the measured temperature (T, K) with the empirical relationship in Equation (3) [31].

(3) $$\eta =0.001\exp\left(0.580-2.520\;\theta +0.909\;\theta^{2}-0.264\;\theta^{3}\right),\;\mathrm{with}\;\theta =\frac{3.661\;\left(T-273.1\right)}{273.1}\;$$

We measured FR as the change in TMP over time during each filtration cycle (dTMP/dt, Pa s^−1^), and calculated it with the linear regression equation presented in Equation (4).

(4) $$FR=\frac{dTMP}{dt}\approx \frac{n\sum_{i=1}^{n}\left(TMP_{i}\;t_{i}\right)-\sum_{i=1}^{n}TMP_{i}\;\sum_{i=1}^{n}t_{i}}{n\sum_{i=1}^{n}t_{i}{}^{2}-{\left(\sum_{i=1}^{n}t_{i}\right)}^{2}}$$

where $t_{i}$ and $TMP_{i}$ are the times and corresponding *TMP* during one filtration cycle, and n is the number of observations.

### 2.7. Statistical Analysis

We compared the mean values from the SMA tests using a Student’s independent t-test assuming equal variances and parametric data. The p was calculated with the *ttest2* function in Matlab^®^ R2019b.

We studied the correlations between sludge characteristics, membrane performance indices and sludge filterability. For the membrane performance indices, we calculated averaged values from a 2-hour period around the sludge sampling time.

Research has proven that biogas or air sparging substantially affects fouling [3,27]. Thus, to eliminate the influence of biogas sparging on membrane performance, we considered only the values of the membrane performance indices when the AnMBR operated under normal biogas sparging, that is, when the modeled ${\mathrm{SGD}}_{m}$ (as calculated with Equation (1)) was between 0.96 and 1.28 Nm^3^ h^−^^1^ m^−2^.

Since the data were not independent for most of the measured variables, we tested the independence of the time-series using a Ljung-Box test, with the function *lbqtest* in Matlab^®^ R2019b (results are not shown). Independence is one of the assumptions of parametric tests; therefore, we used the non-parametric test Spearman’s rank coefficient ($r_{s}$). The statistical significance was assessed by comparing the probability values (p) with a 0.01 level of significance. $r_{s}$ and p were computed with the *corr* function in Matlab^®^ R2019b using “complete” rows (i.e., only rows of the input with no missing values).

## 3. Results

### 3.1. Blackwater and Permeate Characteristics

**Figure 2:**
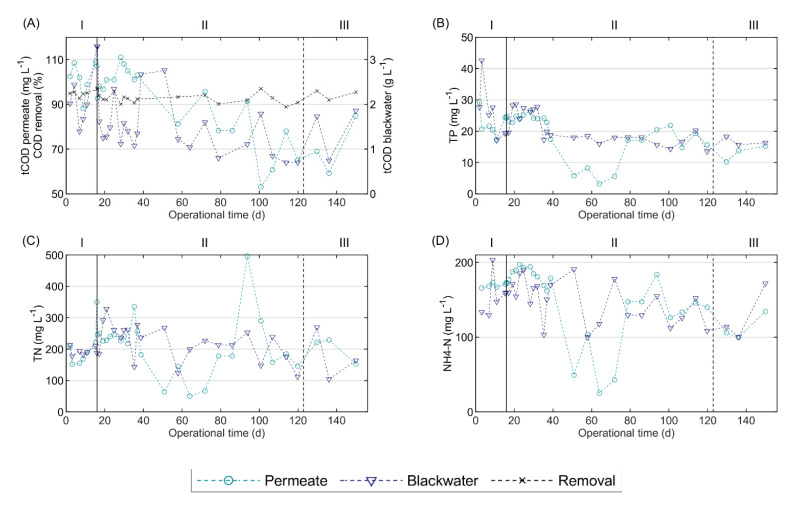
Blackwater and permeate characteristics during the operational period of pilot AnMBR dosed with flux enhancer: (**A**) total COD concentration and COD removal, (**B**) total phosphorous concentration, (**C**) total nitrogen concentration, and (**D**) ammonium-nitrogen concentration. A pulse-dosage of Adifloc KD451 achieving 50 mg L^−1^ was performed on day 16 (black continuous line), and sludge was withdrawn on day 123 (black dotted line). I, II and III are the operational phases described as follows: (I) control phase, (II) period following FE addition, and (III) period following sludge withdrawal.

Figure 2 compares the characteristics of the blackwater and the permeate during all the operational phases; Figure S1 in the Supplementary Materials displays the complete blackwater characterization. The organic matter concentration in the blackwater, measured as tCOD, was highly variable, ranging from 0.7 to 3.3 g L^−1^, and tCOD decreased over time. Because the toilets in the building were conventional gravity flush toilets, as opposed to vacuum toilets, the blackwater tCOD concentration was lower than in other research studies, which reported tCOD values of 8.7 ± 4.0 g L^−1^ [32], 9.8 ± 2.6 and 7.7 ± 2.2 g L^−1^ [33]. High COD removal efficiencies between 89% and 98% were achieved during the entire operational period.

The blackwater characteristics, presented in Figure S1, were highly variable throughout the operational period. This variation may be caused by the small and diverse group of persons generating the blackwater and the lack of external mixing in the septic tank. Approximately 200 persons worked in the building, however the number and specific persons that attended the office varied throughout the week due to the co-working spaces and new companies being installed. Furthermore, the characteristics of the blackwater that was being pumped into the equalization tank were likely affected by the time-of-day and time-of-week that the pumping occurred. For example, the blackwater characteristics may have been different if the equalization tank was filled during office hours, when blackwater was entering the septic tank and thus promoting mixing, as opposed to out-of-office hours, when the septic tank was not mixed and sedimentation was likely to take place.

The concentrations of TN, TP and NH_4_–N in the permeate were similar to those of the blackwater during most of the operational period (Figure 2), because these nutrients are not removed in anaerobic digestion, except for the fraction that is used for biomass growth. Moreover, owing to organic matter mineralization, the NH_4_–N and ortho-phosphate concentrations may even increase in the AnMBR. Nevertheless, during the period of 51 to 72 days, the nutrient concentrations in the permeate were considerably below those of the blackwater, which was possibly caused by increased biomass growth and/or precipitate formation, such as of struvite and calcium phosphate species (Ca_x_(PO_4_)_y_). The increase in blackwater tCOD load in the mentioned period might have led to increased biomass growth, agreeing with the observed increase in VSS concentration (Figure S2E). The concomitant nutrient requirements for biomass growth would then result in decreased NH_4_–N and TP concentrations in the permeate, as was shown in Figure 2. The estimated requirements of nitrogen and phosphorous for biomass growth when the blackwater tCOD increased to 2.7 gCOD L^−1^ were 14–19 mg N and 1–4 mg P per litre of influent, respectively. These values were calculated assuming a biomass yield of 0.10, a COD conversion of 92%, a biomass COD to VSS conversion of 1.42 gCOD gVSS^−1^, and a nitrogen and phosphorous requirement based on the elemental composition of biomass, namely 80.8–108.8 mgN mgVSS^−1^ and 4.3–23.8 mgP gVSS^−1^ [34]. However, the observed decrease in NH_4_–N and TP from blackwater to permeate (Figure 2) largely exceeded the calculated biomass growth-related values, and amounted to 92–142 mgN L^−1^ and 10–13 mgP L^−1^, respectively. Therefore, the decreased NH_4_–N and TP concentrations in the permeate were likely caused by precipitate formation, which is influenced by the environmental conditions, such as pH and the concentrations of different ions, in the reactor. Particularly, the precipitation of Ca_x_(PO_4_)_y_ has been observed in reactors treating blackwater [35,36].

During the 10-day periods before and after FE addition, the mean COD removals were 94.8% and 94.2%, and the mean permeate tCOD values were 94.8 and 94.2 mg L^−^^1^, respectively. Therefore, COD removal and permeate tCOD were seemingly not affected by the addition of FE. Furthermore, around the moment of FE addition, the TN, TP and NH_4_–N levels were increasing in the blackwater, and consequently in the permeate, because these nutrients are not removed during anaerobic digestion. Therefore, the increased concentrations in the permeate were very likely caused by their increase in blackwater, and not by FE addition.

The sludge withdrawal from the AnMBR performed on day 123 removed 64% of VSS. The huge drop in VSS concentration did not impact COD removal or permeate quality in terms of COD concentration. Apparently, the potential organic loading or volumetric conversion capacity of the AnMBR was not fully utilized.

### 3.2. Sludge Characteristics

**Figure 3:**
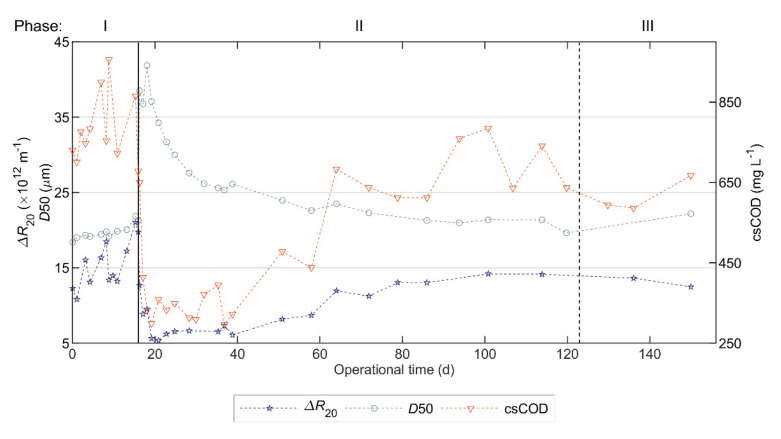
Sludge characteristics during the operational period of the pilot AnMBR dosed with flux enhancer: filterability expressed as $∆R_{20}$ ($∆R_{20}$ is inversely related with filterability), floc size expressed as 50th percentiles of volume-based particle size distribution (D50 ), and submicron COD concentration (csCOD). A pulse-dosage of Adifloc KD451 achieving 50 mg L^−1^ was performed on day 16 (black continuous vertical line), and sludge was withdrawn on day 123 (black dotted vertical line).

Figure 3 shows the most relevant sludge characteristics, and Figure S2 in Supplementary Materials contains the complete characterization.

During days 13 to 18, the sludge recirculation pump (P-3 in Figure 1) malfunctioned and caused an accumulation of solids in the membrane tank, shown by the increased TSS and VSS in Figure S2D,E, respectively. The pump was repaired on day 18, and the TSS returned to its original value. Furthermore, as explained in Section 3.1, a rapid increase in VSS was observed from days 39 to 51 (Figure S2E), which we attributed to an increased blackwater tCOD (Figure 2A) that promoted biomass growth and the accumulation of un-degraded particulate organic matter. Figure S2E shows a similar rapid VSS increase on day 94; however, on this occasion, the sudden increase cannot be explained by a high blackwater tCOD. Instead, the increased VSS was possibly caused by the accumulation of un-degraded particulate or colloidal organic matter from the blackwater, as a result of a change in blackwater composition, which we did not notice when using the applied physicochemical characterization. Additionally, the lower temperature (Figure S4A) could have reduced the hydrolysis rate, concomitantly explaining the slight decrease in COD removal efficiency (Figure 2A).

During the 10-day periods before and after FE addition, the mean $∆R_{20}$ values were 16.7 × 10^12^ and 7.8 × 10^12^ m^−1^, the mean D50 values were 20.2 and 35.7 µm and the mean csCOD values were 740 and 391 mg L^−1^, respectively. Therefore, on average, FE addition decreased the $∆R_{20}$ value by 53% (i.e., improved sludge filterability), increased D50 by 77% and decreased csCOD by 47%.

During Phase II, the effect of FE on filterability decreased slowly—the $∆R_{20}$ value increased on average 0.1 × 10^12^ m^−1^ per day. The filterability stayed below the lowest registered value in Phase I (i.e., 10.8 × 10^12^ m^−1^) for a 50-day period, and achieved values similar to the mean $∆R_{20}$ in Phase I (i.e., 14.2 × 10^12^ m^−1^) after 85 days. Furthermore, the increase in $∆R_{20}$ was simultaneous with the csCOD increase and D50 decrease.

The sludge withdrawal from the AnMBR, whereby 31% of the liquid volume was removed on day 123, caused a 62% decrease in TSS and only a 7% decrease in csCOD, and a 4% decrease in $∆R_{20}$. The high decrease in TSS and low decrease in csCOD likely can be attributed to the fact that the purge was done from the bottom of the membrane tank, where particulate material is deposited by sedimentation, while colloidal material remains suspended.

### 3.3. SMA Tests

**Figure 4:**
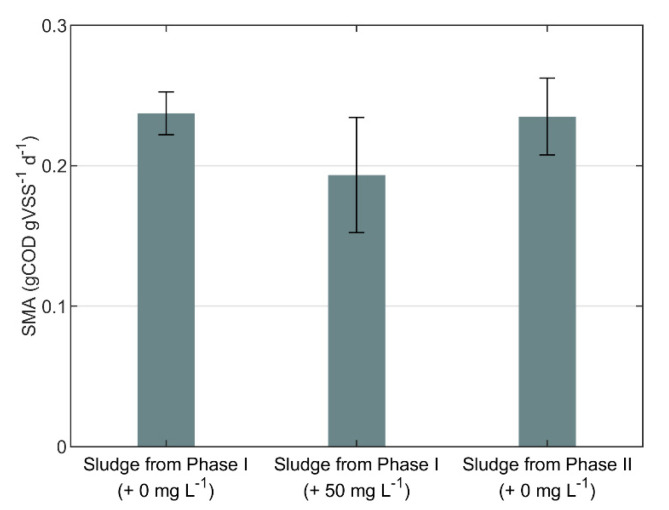
Specific methanogenic activity (SMA) of sludge samples collected from the pilot AnMBR immediately before (Phase I) and 3 weeks after (Phase II) FE addition. The values between brackets are the concentrations of Adifloc KD451 added to the SMA bottles, pre-mixed with inoculum. The error bars are the 95% confidence intervals.

The effect of Adifloc KD451 on the biological activity was assessed with SMA tests, and the results are summarized in Figure 4. For Inoculum I, the mean SMA with 50 mg L^−1^ of Adifloc KD451 added to the bottle was 18% lower than the SMA without FE addition; this difference was statistically significant, with p=0.012. Moreover, the mean SMA of Inoculum II did not present a statistically significant difference, p=0.76, from the mean SMA of Inoculum I.

### 3.4. $SGD_{m}$ Model Calibration

**Figure 5:**
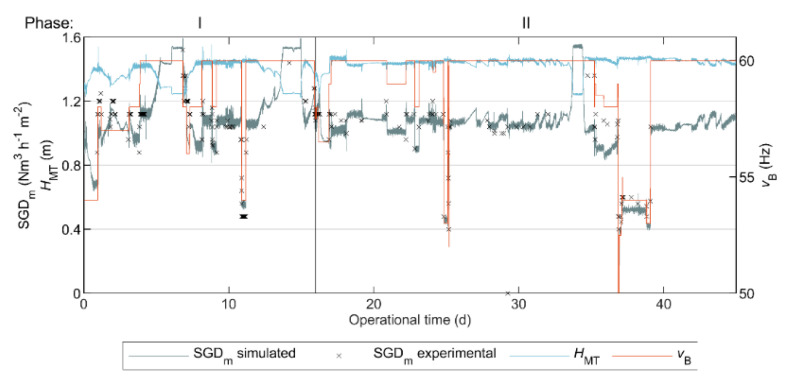
Simulated and experimental specific gas demand (${\mathrm{SGD}}_{m}$), level of liquid in the membrane tank ($H_{\mathrm{MT}}$) and motor frequency of the blower ($v_{B}$) during the operational period of the AnMBR dosed with flux enhancer.

The estimated parameters of the ${\mathrm{SGD}}_{m}$ model in Equation (1), to evaluate the required gas sparging demand, were as follows:$\;\beta_{0}=-3.43$ Nm^3^ h^−^^1^; $\beta_{1}=-14.57$ Nm^3^ h^−^^1^ m^−^^1^; and $\beta_{2}=0.52$ Nm^3^ h^−^^1^ Hz^−^^1^. Figure 5 shows the experimental and simulated ${\mathrm{SGD}}_{m}$. The Pearson correlation coefficient between the experimental and simulated results was 0.906. Thus, the proposed model satisfactorily predicted ${\mathrm{SGD}}_{m}$, and therefore, the simulated ${\mathrm{SGD}}_{m}$ could be used as a continuous estimation of ${\mathrm{SGD}}_{m}$ in the pilot AnMBR.

As described in Section 2.7, we used the continuous simulated ${\mathrm{SGD}}_{m}$ to eliminate the influence of biogas sparging on membrane fouling when studying the correlation between sludge characteristics, membrane performance indices and sludge filterability.

### 3.5. AnMBR Filtration Performance

**Figure 6:**
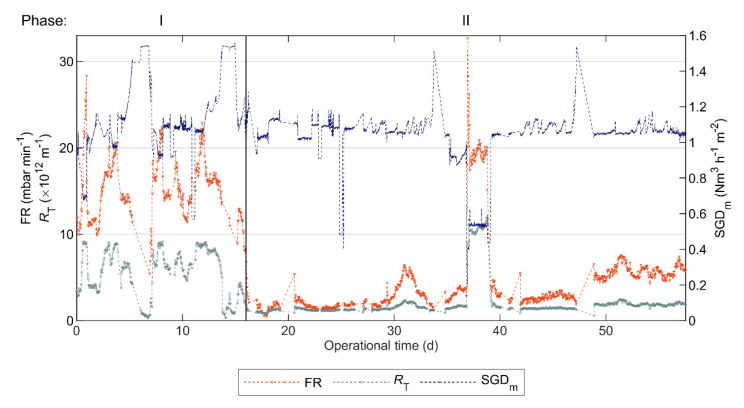
Mean hourly fouling rate (FR), mean hourly total filtration resistance ($R_{T}$), and simulated specific gas demand (${\mathrm{SGD}}_{m}$) in pilot AnMBR dosed with flux enhancer. A pulse-dosage of Adifloc KD451 achieving 50 mg L^−1^ was performed on day 16 (black continuous vertical line).

The filtration performance of the AnMBR was assessed based on FR and $R_{T}$, as shown in Figure 6. Figures S3 and S4 in the Supplementary Materials display the other AnMBR variables recorded by the SCADA. The TMP could not be measured after day 58 due to technical difficulties with the on-line measurement of permeate pressure, that could not be resolved; thus the $R_{T}$ and FR could not be calculated after day 58.

During the 10-day periods before and after FE addition, the mean $R_{T}$ values were 6.6 × 10^12^ and 1.2 × 10^12^ m^−1^ and the mean FR values were 15.3 and 1.7 mbar min^−1^, respectively. Therefore, FE addition improved the filtration performance of the AnMBR, clearly indicated by an 82% mean $R_{T}$ decrease and an 89% mean FE decrease. Furthermore, during the 42-day period recorded by the SCADA in Phase II (Figure 6), the FR and $R_{T}$ values remained below the ones registered during Phase I.

The biogas blower did not have an automatic control, but instead was operated at a fixed motor frequency set by the operator. Thus, the ${\mathrm{SGD}}_{m}$ varied with the pressure in the membrane tank, which was determined by $H_{\mathrm{MT}}$. Since the AnMBR was fed with blackwater generated in an office building, mostly empty outside working hours, there were weekends when there was less blackwater production (mainly during Phase I) and the reactor was not fed, resulting in decreased $H_{\mathrm{MT}}$ and increased ${\mathrm{SGD}}_{m}$, as seen in Figure 5. Consequently, during these periods of low $H_{\mathrm{MT}}$, the resulting FR and $R_{T}$ values were low. Accordingly, FR and $R_{T}$ considerably increased with decreasing ${\mathrm{SGD}}_{m}$ in Phase II (Period: 37–39 d). Therefore, as expected, the filtration performance on the AnMBR improved (in the short-term) with the higher biogas sparging rate, and conversely, it deteriorated with the low sparging rate.

### 3.6. Correlation Analysis

Table 1 shows that the csCOD and D50 had statistically significant correlations with the $∆R_{20}$, $R_{T}$ and FR. The correlation coefficients were negative for D50 and positive for csCOD, suggesting that a sludge with a higher D50 and lower csCOD had better filterability and created less fouling. The results show that the D50 and csCOD were statistically significantly correlated.

## 4. Discussion

### 4.1. Potential Foulants and the Role of Flux Enhancer

#### 4.1.1. Soluble and Colloidal Organic Matter

When added to a sludge sample, cationic polymers such as Adifloc KD451 are adsorbed onto the negatively charged surface of the suspended organic matter, such as colloidal material and flocs, promoting their agglomeration upon collision, consequently decreasing the concentration of colloidal material and increasing particle size. Moreover, colloidal and soluble material could be incorporated into the flocs by interaction with the cationic polymer adsorbed, or by entrapment between aggregated flocs. Accordingly, the addition of Adifloc KD451 to the AnMBR considerably decreased csCOD, which comprises colloidal and soluble material, and increased floc size.

The correlation analysis, in Section 3.6, showed that although both csCOD and D50 correlated with $∆R_{20}$, FR and $R_{T}$, it was not possible to elucidate the individual effect of D50 and csCOD on fouling and filterability. Nevertheless, in a previous study [24], $∆R_{20}$ statistically significantly correlated with csCOD, cCOD and sCOD, but not with D50. Accordingly, in Iversen [8], four of the tested FEs had no significant effect on mean floc size, while they decreased SMP and improved critical flux. Zhang et al. [25] dosed 100 mg L^−1^ polyaluminium chloride or 400 mg L^−1^ PAC-SAE-Super, which reduced the TMP in a cross-flow filtration cell, while the mean floc size did not change significantly, and both colloidal and soluble organic matter decreased. Therefore, the results from previous research suggest that colloidal and soluble organic matter removal might have a higher impact on fouling mitigation than increasing floc size. Accordingly, researchers have consistently identified colloidal material as a major factor in reversible fouling in membrane bioreactors [3,19,37,38,39]. High colloidal concentrations increase the fouling rate by cake layer formation, pore blocking, and decreasing cake layer porosity.

#### 4.1.2. Floc Size

The addition of Adifloc KD451 to the AnMBR considerably increased median floc size, measured as D50. The effect of floc size on fouling has been addressed by several authors. Larger flocs can form more porous cakes, reduce the adhesion of the flocs to the membrane, increase the back-transport of flocs from the membrane surface to the bulk liquid, and reduce cake layer thickness by surface erosion or increased shear near the membrane surface [37,40,41,42], consequently decreasing membrane fouling.

Accordingly, the correlation analysis suggested that a higher D50 increased the sludge filterability and created less fouling. However, in Figure 3, the changes in D50 were simultaneous with, and opposite to, the changes in csCOD. Similarly, different researchers observed a decrease in fouling rate when simultaneously increasing the mean floc size and decreasing the SMP or the concentration of submicron particles [8,43,44,45,46,47,48]. To the best of our knowledge, Zhang et al. [25] is the only study wherein the fouling rate decreases with an increasing floc size, while no removal, or even a slight increase, of colloidal particles or SMP was observed. Therefore, from these studies and our results, it is challenging to elucidate to what extent, if any, floc size affected fouling, or if the fouling improvement was simply caused by soluble and colloidal organic matter removal. Furthermore, some studies suggest that floc size had no effect on fouling mitigation, or even had a negative effect, which the author attributed to a decrease in the extracellular polymeric substances (EPS) [49] and to changes in the structure of the flocs [19]. Nevertheless, the Carman-Kozeny equation and thermodynamic approaches suggest that floc size directly affects membrane fouling, and that small flocs have a stronger effect than large flocs [40,41]. Therefore, floc size might have a significant effect on membrane fouling for small flocs, whereas further increasing the size of already large particles might have a negligible effect on fouling mitigation.

The stronger correlation between D50 and filtration performance in the AnMBR (i.e., FR and $R_{T}$) than between D50 and filterability (Table 1) might be due to the different membrane configuration and operational conditions in the AnMBR compared to the AnDFCm installation, such as hydrodynamic conditions, membrane type, filtration and relaxation cycles, and mechanical cleaning (gas sparging vs. liquid cross-flow). Particularly, the AnMBR operates with filtration and relaxation cycles, whereas the AnDFCm installation operates with continuous filtration during filterability measurements. A recent study has demonstrated that under laminar conditions and without applying a membrane flux, large particles tend to move in larger numbers to the membrane than smaller particles, and with a membrane flux, large and small particles move in similar numbers [50]. Considering this, during relaxation in the AnMBR, the migration of larger particles to the membrane could create a cake layer with a high number of large particles, contributing to a highly permeable cake that can act as a protective layer during the filtration phase. Contrarily, the AnDFCm installation worked continuously in the filtration mode, and consequently this hypothetical protective cake layer formed by large particles was not formed, and thus the size of the large particles was less relevant.

### 4.2. Extent of Flux Enhancer Effect

FE addition had a long-term effect on sludge filterability and filtration performance; nevertheless, those effects slowly deteriorated over time, as shown by the $∆R_{20}$ returning to similar values as in Phase I, 85 days after FE addition. Similarly, after a pulse-dosage of MPE50 to a pilot MBR (R11 in Table S1), the MBR operated with a higher flux and a slightly lower TMP compared to the control phase during a 30-day period, after which sludge was withdrawn and the filtration performance deteriorated [17]. Moreover, Diaz et al. [21] achieved a higher flux during a 4-week operating period compared to the control phase, after dosing MPE50 to a lab-scale AnMBR fed with synthetic wastewater. During this period no sludge was withdrawn.

Therefore, despite operating without sludge wastage, the effect of the FE can be lost over time due to several reasons, such as the following: biomass and colloidal material accumulation in the reactor due to microbial growth, floc breakage or the detachment of particulate material from flocs caused by shear; the loss of FE in the permeate or by FE biodegradation; and changes in sludge characteristics caused by fluctuations in the operational conditions or substrate characteristics.

### 4.3. Flux Enhancer Effect on Permeate Quality

When FE is added to sludge, a fraction of FE can remain unbounded in the bulk liquid, depending on a physicochemical equilibrium. Previous research [24] showed that Adifloc KD451 can pass through the membrane pores of the AnDFCm and contaminate the permeate. The membrane in the AnMBR had similar nominal pore size to the AnDFCm installation, that is, 35 and 30 nm, respectively. Thus, if the FE remains unbounded it can contaminate the AnMBR permeate. The AnMBR was dosed with 50 mg L^−1^ of Adifloc KD451, and thus assuming that 5% of the added FE remained unbounded [51] and reached the permeate, that is 2.5 mg L^−1^ or 2.8 mgCOD L^−1^. If this had been the case in our current experiment, and considering that the AnMBR permeate COD was between 90 and 110 mg L^−1^, the contribution of FE to the permeate COD would have been negligible. Accordingly, the results in Section 3.1 show that the permeate COD was not affected by the addition of FE. Furthermore, the permeate nutrient concentrations, namely TP, TN and NH_4_–N, were apparently not affected by the FE addition. Therefore, the addition of Adifloc KD451 to the AnMBR had seemingly no effect on permeate quality. Accordingly, MPE50 had no detrimental effect, or even slightly improved the permeate quality and nutrient removal [9,14,16,17]. Therefore, the addition of FE, particularly cationic polymers, to MBRs and AnMBRs has no significant adverse effect on permeate quality.

### 4.4. Flux Enhancer Effect on Biological Activity

Under anaerobic conditions, our results showed that Adifloc KD451 had an immediate inhibitory effect on the biological activity in the SMA test, which was in accordance with previous observations [52]. Contrarily, under aerobic conditions, different cationic polymers, including Adifloc KD451, had no detrimental effect on the oxygen uptake [53,54].

The SMA of sludge collected 3 weeks after FE addition to the AnMBR did not present a statistically significant difference from the sludge collected immediately before FE addition. Therefore, as proposed in previous research [52], although Adifloc KD451 had an immediate inhibitory effect on SMA, this was regarded as a reversible process. Moreover, the applied organic sludge loading rates to the AnMBR were relatively low, i.e., 0.01–0.18 gCOD gVSS^−1^ d^−1^, meaning that a reduction of 18% in SMA does not harm the process. It should be noted that in addition to the observed effect on SMA, FE may also irreversibly bind organic matter, reducing the biomethane potential of the substrate [55] and thus the overall biogas production rate. Unfortunately, the daily biogas production could not be measured in our current pilot experiment.

The results clearly show that FE addition had no adverse effect on COD removal in the AnMBR, as presented in Section 3.1, which is in accordance with previous research performed with MPE50 [14,21]. Therefore, our research suggests that the addition of FE, particularly cationic polymers, has no significant adverse effect on COD removal efficiencies.

### 4.5. Sludge Withdrawal as an Alternative Strategy for Fouling Control

As shown in Section 3.2, the sludge withdrawal performed on day 123 caused a high decrease in TSS (62% decrease), while it only slightly decreased csCOD (7% decrease), which was likely attributable to the location of the purge (in the bottom of the membrane tank) and the amount of withdrawn reactor broth. The low decrease in csCOD caused a small improvement in sludge filterability ($∆R_{20}$ decreased 4%). The results indicate that sludge withdrawal is not a very effective fouling control strategy, as the major part of csCOD remains suspended in the bulk of the liquid. Likely, only very large volume exchange ratios will impact the bulk liquid csCOD concentrations and thus the total membrane resistance. We did not perform further experiments to prove this hypothesis since it was outside the scope of this paper.

### 4.6. Filterability as Input Variable for Fouling Control and Flux Enhancer Dosing

Under the normal operational biogas sparging conditions defined in Section 2.1, i.e., ${\mathrm{SGD}}_{m}$ = 0.96–1.28 Nm^3^ h^−1^ m^−2^, the sludge filterability was statistically correlated with the AnMBR filtration performance indices (i.e., FR and $R_{T}$), and thus the observed improvement in AnMBR filtration performance was possibly due to the improved sludge filterability. However, during the period of 37–39 days, the AnMBR filtration performance deteriorated, while sludge filterability slightly improved, and conversely, the AnMBR filtration performance improved while sludge filterability deteriorated during the periods of day 6–7 and day 13–14. These behaviors were attributed to changes in ${\mathrm{SGD}}_{m}$; see Section 3.5. Therefore, relating sludge filterability to AnMBR filtration performance indices allowed us to identify the cause of filtration performance deterioration or improvement in the AnMBR.

Consequently, a fouling control strategy that uses sludge filterability and AnMBR filtration performance indices as input variables could help decide on the appropriate countermeasure [56], that is, manipulate either the sludge characteristics, for example by adding FE, or the membrane operational conditions, for example by decreasing flux or increasing biogas sparging. Furthermore, the AnDFCm takes 20 min to determine filterability, and this time is negligible compared to the rate of change of filtration performance and sludge filterability observed in membrane bioreactors, as shown in Section 4.2. This means that the dynamic of the measuring sensor is considerably faster than the dynamics in the process parameters, which is an indispensable property in a successful control strategy. Therefore, in-situ filterability measurements with the AnDFCm proved to be an appropriate input variable for manipulating FE dosage for fouling control in AnMBRs.

## 5. Conclusions

This research evaluated the long-term effect on filtration performance, sludge characteristics, biological activity and permeate quality following a pulse-dosage of Adifloc KD451 as FE to a concentration of 50 mg L^−^^1^ in a pilot AnMBR. The main findings can be summarized as follows:

- FE addition improved the filtration performance of the AnMBR, as indicated by the 82% $R_{T}$ and 89% FR reductions, without significantly affecting COD removal and permeate quality. The improvement was sustained in the long term—the FR and $R_{T}$ values stayed below the ones registered during the control phase (i.e., Phase I) for at least 42 days, and after this period the SCADA system failed to measure FR and $R_{T}$.
- The improved filtration performance was attributed to increased floc size and reduced csCOD (i.e., colloidal + soluble organic matter concentration), thereby improving sludge filterability. The filterability returned to similar values as in the control phase 85 days after FE addition.
- The SMA values of the sludge samples collected immediately before and 3 weeks after FE addition were statistically similar; however, in batch tests, 50 mg L^−1^ of Adifloc KD451 caused an 18% SMA inhibition. Thus, the FE had a modest immediate effect on the SMA, which, however, had no significant impact on the AnMBR performance. Moreover, the drop in SMA was reversible.
- Relating in-situ measurements of sludge filterability with AnMBR filtration performance indices, i.e., FR and $R_{T}$, allowed us to identify the prevailing gas sparging rate as being the cause of filtration performance deterioration or improvement in the AnMBR.
- In-situ measurements of sludge filterability with the AnDFCm proved an appropriate input variable for manipulating FE dosage for fouling control in AnMBRs.

## Figures and Tables

**Table 1:** Spearman correlation half matrix between sludge characteristics, total filtration resistance ($R_{T}$ ), fouling rate (FR) and sludge filterability (expressed as $∆R_{20}$ ). Significant correlation at level 0.01 (*); probability value (p) given between brackets.

Variable	$R_{T}$	FR	$∆R_{20}$	csCOD	D50
$R_{T}$	1				
FR	0.96 * (8 × 10^−12^)	1			
$∆R_{20}$	0.64 * (8 × 10^−5^)	0.75 * (8 × 10^−5^)	1		
csCOD	0.75 * (9 × 10^−5^)	0.82 * (5 × 10^−6^)	0.82 * (5 × 10^−6^)	1	
D50	−0.89 * (8 × 10^−8^)	−0.84 * (2 × 10^−6^)	−0.58 * (6 × 10^−3^)	−0.74 * (1 × 10^−4^)	1

## Supplementary Materials

The following are available online at https://www.mdpi.com/2073-4360/12/10/2383/s1, Table S1: Literature review of flux enhancers (FE) applied to pilot and full-scale MBRs and AnMBRs fed with real wastewater, Figure S1: Blackwater characteristics during operational period of pilot AnMBR plant dosed with flux enhancer, Figure S2: Sludge characteristics during operational period of pilot AnMBR plant dosed with flux enhancer, Figure S3: Pilot AnMBR mean hourly membrane performance state variables during operational period of pilot AnMBR plant dosed with flux enhancer, Figure S4: AnMBR mean hourly mixed liquor state variables during operational period of pilot AnMBR plant dosed with flux enhancer.
