# Supplementary material for: Fouling Mitigation by Cationic Polymer Addition into a Pilot-Scale Anaerobic Membrane Bioreactor Fed with Blackwater
Source: Polymers (Basel). 2020 Oct 16;12(10):2383. doi: 10.3390/polym12102383 (PMC7602741; doi:10.3390/polym12102383)
Supplement: Supplementary file 1 [file polymers-12-02383-s001.pdf]

polymers

Supplementary material

Fouling Mitigation by Cationic Polymer Addition into a Pilot-Scale Anaerobic Membrane Bioreactor Fed with Blackwater

Magela Odriozola [1,*], Nicolás Morales [2], Jose R. Vázquez-Padín [2], Maria Lousada-Ferreira [1,†], Henri Spanjers [1] and Jules B. van Lier [1].

[1] Department of Water Management, Delft University of Technology, Stevinweg 1, 2628 CN Delft, the Netherlands; M.OdriozolaArbiza@tudelft.nl (M.O.); M.LousadaFerreira@tudelft.nl (M.L.-F.); H.L.F.M.Spanjers@tudelft.nl (H.S.); J.B.vanLier@tudelft.nl (J.v.L.)
[2] Aqualia, Rúa das Pontes 4, 36350 Nigrán, Pontevedra, Spain; nicolas.morales.pereira@fcc.es (N.M.); jvazquezp@fcc.es (J.V.-P.)
† Present address: KWR Water Research Institute, Groningenhaven 7, 3430 BB Nieuwegein, the Netherlands.
* Correspondence: M.OdriozolaArbiza@tudelft.nl.

Table of contents

S1. Supporting material: literature review table 2

S2. Supporting material: pilot AnMBR plant monitoring 3

S3. References 6

S1. Supporting material: literature review table

Table S1. Literature review of flux enhancers (FE) applied to pilot and full-scale MBRs and AnMBRs fed with real wastewater.

Reactor reference	FE type [a], dosage ($mg\ L^{-1}$)	Dosing control [b]	Dosing strategy [c]	Reactor type	Membrane surface area (m^2)	Reactor volume (m^3)	Wastewater (fed)	Operational time with FE (d)	Main effects of FE presence [g]	Reference
R1	$FeCl_3$, 26	FF	Step in influent	AnMBR	5.4	0.55	Municipal	90	↓fouling, ↑thickness and porosity fouling layer, ↓PR and PS on membrane surface, ↑COD removals, ↓colloidal COD ↓soluble COD, ↑particle size	[1]
R2	$FeCl_3$, 12-21, 43	FF	Step in influent	AnMBR	5.4	0.55	Municipal	70	↓reversible fouling, ↓colloidal COD, ↓VSS biodegradability	[2]
R3	PACl, 12.5 mg $gTSS^{-1}$	FF	Pulse + compensation (W)	MBR	1	-	Mixed domestic and textile	65	↓fouling rate, ↓cake resistance, ↑filtration stability, ↕SMP-PR, ↕SMP-PS, ↓extracted EPS-PR and EPS-PS	[3]
R4	Mylbond168, 1,500-2,000	FF	Pulse + compensation (W)	MBR	22	1	Municipal	50	↑TMP, washed out with permeate, ↑floc size, ↓CST	[4]
R4	KD452, 70	FF	Pulse + compensation (W)	MBR	22	1	Municipal	63	↓TMP, retarded fouling, ↕nutrient removal, ↓SMP, ↑floc size, ↓biopolymers	[4]
R4	MPE50, 500	FF	Pulse + compensation (W)	MBR	22	1	Municipal	74	↓TMP, retarded fouling, ↕nutrient removal, ↓SMP, ↑ floc size, ↓CST	[4]
R5	MPE50, 250	FF; FB	Pulse + compensation (W & B); Pulses	MBR	0.9	0.12	Refinery effluent	220	↓fouling resistance, ↓TTF, ↑PSD, ↓colloidal TOC, ↓SMP, ↓EPS	[5]
R6	MPE50, 400	FF	Pulse + compensation (B)	MBR	60	10.2	Municipal	20	↑flux, ↓TMP, ↓foam	[6]
R7	MPE50, 400	FF	Pulse + compensation (unspecified)	MBR	1000	-	Leachate	30	↓TMP, ↑permeability, ↑flow, ↓permeate COD, ↓chemical cleaning frequency, ↓foam	[7]
R8	MPE50, 600	FF	Pulse + compensation (unspecified)	MBR	-	125	Food Industry	12	↓TMP, ↑permeability, ↓ permeate COD, ↓chemical cleaning frequency, ↓foam	[7]
R9	MPE50, 500	FF	Pulse + compensation (unspecified)	MBR	0.5	0.2	Municipal	14	↑critical flux	[8]
R10	MPE50, 400	FF	Pulse + compensation (unspecified)	MBR	-	750 [d]	Municipal	>1 [f]	↑permeability, ↑one-day peak flux	[8]
R11	MPE50, 200	FF	Pulse	MBR	6.4	-	Municipal	35	↑flux, ↓permeate COD	[8]
R12	MPE50, 300	FF	Pulse + compensation (W)	MBR	-	- [e]	Municipal with hydrophilic waxes	35	↑flux, ↓shuts down triggered by high TMP, ↑one-day peak flux	[8]
R13	PAC, 1,500, 3,000	FF	Pulse + compensation (unspecified)	MBR	6	0.52	Tannery Industry	139	↓fouling rate, ↓chemical cleanings, COD removal stabilization	[9]
R14	PAC, 500	FF	Ramp up until desired concentration	MBR	0.1	0.085	Municipal	140	↑critical flux, ↑sustainable filtration period, ↓gel-cake deposition, ↑removability of gel-cake, ↑permeate quality	[10]

[a] MPE50 and KD452 are cationic polymers; PACl is polyaluminum chloride; and Mylbond168 is a starch.

[b] FF: feedforward dosing, where FE is dosed to achieve a target concentration. FB: feedback dosing, where FE is added based on the value of an input variable.

[c] Pulse: reactor spiked with FE. Pulse + compensation: initial pulse-dosage followed by periodic additions to compensate loss of FE with sludge withdrawal (W) and 1% biodegradable fraction (B).

[d] Total bioreactor volume calculated based on total MPE50 added (300 kg) and target concentration (400 $mg\ L^{-1}$).

[e] Design operational volume 50 $m^3\ d^{-1}$.

[f] Reported results for 1 day, but MPE50 remained in the system.

[g] Nomenclature:↑, increase; ↓, decrease; ↕, no significant change. Abbreviations: COD, chemical oxygen demand; CST, capillary suction time; EPS, extracellular polymeric substances; PR, proteins; PS, polysaccharides; PSD, particle size distribution; SMP, soluble microbial products; TMP, transmembrane pressure; TTF, time-to-filter; VSS, volatile suspended solids.

S2. Supporting material: pilot AnMBR plant monitoring

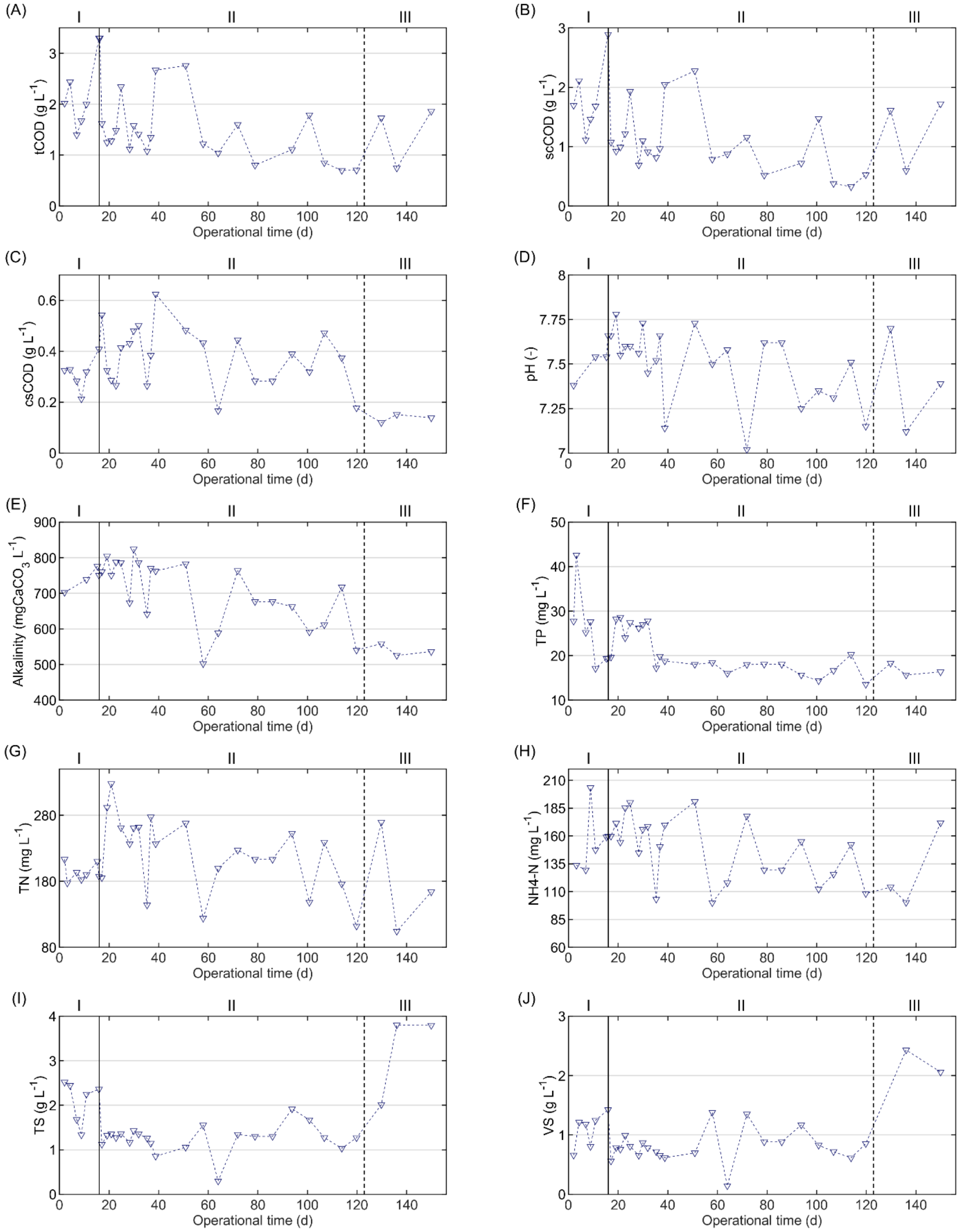

Figure S1. Blackwater characteristics during operational period of pilot AnMBR plant dosed with flux enhancer: (A) total COD, (B) supracolloidal COD, (C) submicron COD, (D) pH, (E) alkalinity, (F) total phosphorous, (G) total nitrogen, (H) ammonium-nitrogen, (I) total solids, and (F) volatile solids.

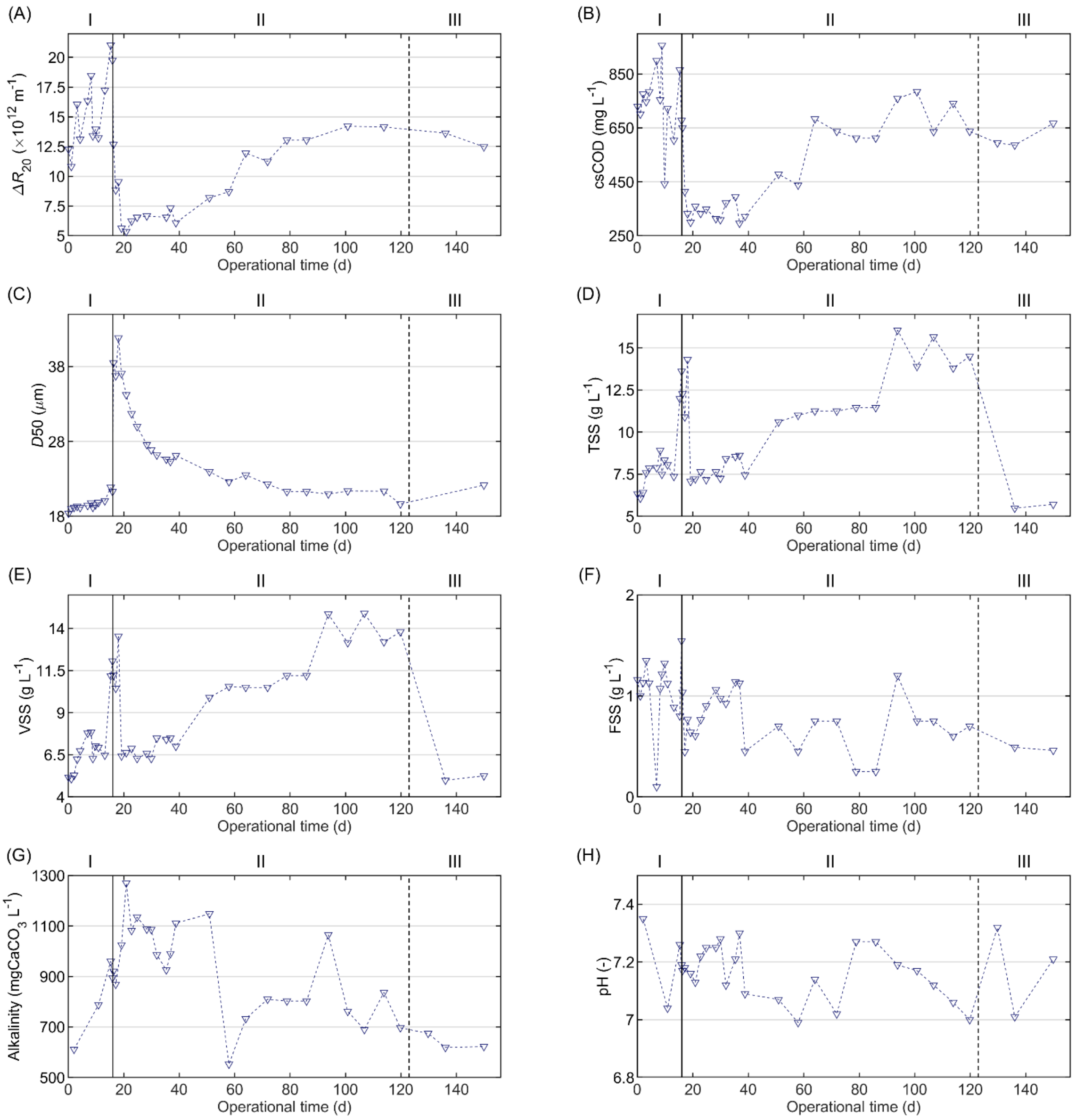

Figure S2. Sludge characteristics during operational period of pilot AnMBR plant dosed with flux enhancer: (A) sludge filterability expressed as ΔR_{20} (which is inversely related with filterability), (B) submicron COD, (C) floc size expressed as 50th percentiles of volume-based particle size distribution, (D) total suspended solids, (E) volatile suspended solids, (F) fixed suspended solids, (G) alkalinity, and (H) pH.

We calculated the mean TMP (TMP_{ave}, Pa) as the average of the TMP values recorded by the SCADA system during one filtration cycle (TMP_i), as follows:

$$TMP_{ave} = \frac{\sum TMP_i}{n} \quad (S1)$$

, where n is the number of observations.

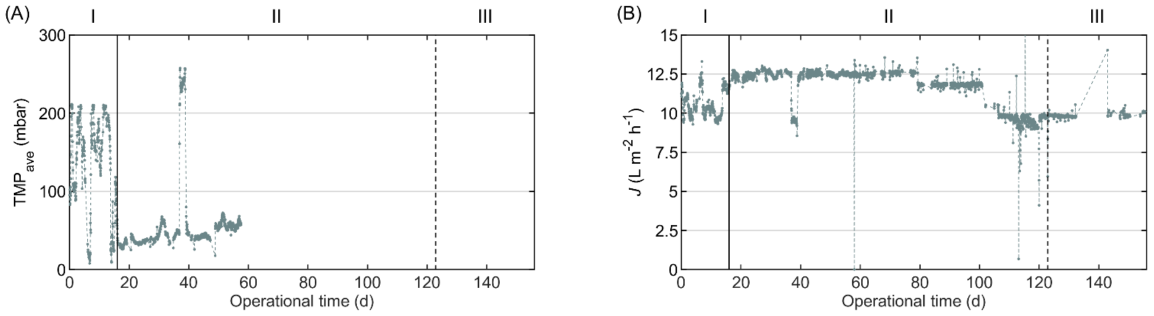

Figure S3. Pilot AnMBR mean hourly membrane performance state variables during operational period of pilot AnMBR plant dosed with flux enhancer: (A) mean TMP during one filtration cycle, and (B) transmembrane flux.

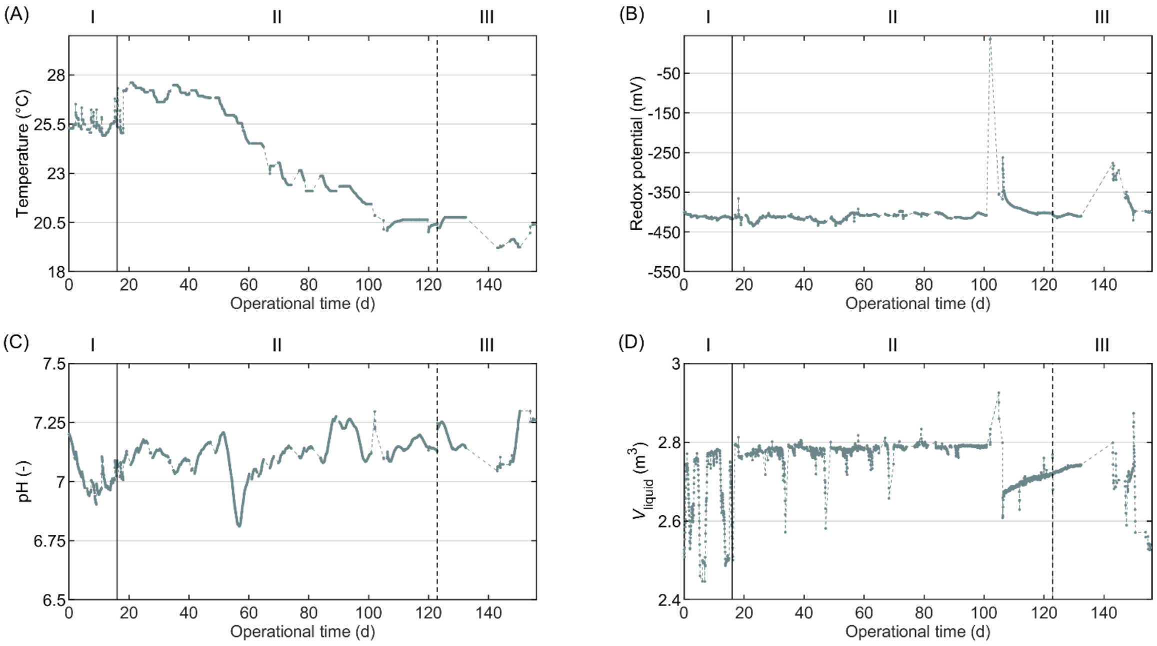

Figure S4. AnMBR mean hourly mixed liquor state variables during operational period of pilot AnMBR plant dosed with flux enhancer: (A) temperature, (B) redox potential, (C) pH, (D) total liquid volume (membrane tank + anaerobic reactor).

S3. References

1. Dong, Q.; Parker, W.; Dagnew, M. Impact of $FeCl_3$ dosing on AnMBR treatment of municipal wastewater. *Water Res.* **2015**, *80*, 281–93, doi:10.1016/j.watres.2015.04.025.
2. Dong, Q.; Parker, W.; Dagnew, M. Dynamic characterization of a FeCl3-dosed anaerobic membrane bioreactor (AnMBR) treating municipal wastewater. *Water Sci. Technol.* **2018**, *2017*, 481–491, doi:10.2166/wst.2018.175.
3. Teli, A.; Antonelli, M.; Bonomo, L.; Malpei, F. MBR fouling control and permeate quality enhancement by polyaluminium chloride dosage: a case study. *Water Sci. Technol.* **2012**, *66*, 1289–1295, doi:10.2166/wst.2012.315.
4. Iversen, V.; Mehrez, R.; Horng, R.Y.; Chen, C.H.; Meng, F.; Drews, A.; Lesjean, B.; Ernst, M.; Jekel, M.; Kraume, M. Fouling mitigation through flocculants and adsorbents addition in membrane bioreactors: Comparing lab and pilot studies. *J. Memb. Sci.* **2009**, *345*, 21–30, doi:10.1016/j.memsci.2009.08.014.
5. Alkmim, A.R.; da Costa, P.R.; Moser, P.B.; França Neta, L.S.; Santiago, V.M.J.; Cerqueira, A.C.; Amaral, M.C.S. Long-term evaluation of different strategies of cationic polyelectrolyte dosage to control fouling in a membrane bioreactor treating refinery effluent. *Environ. Technol.* **2016**, *37*, 1026–1035, doi:10.1080/09593330.2015.1096964.
6. Collins, J.H.; Yoon, S.-H.; Musale, D.; Kong, J.F.; Koppes, J.; Sundararajan, S.; Tsai, S.-P.; Hallsby, G.A.; Cachia, P.; Kronoveter, K. Membrane performance enhancer evaluations on pilot- and full-scale membrane bioreactors. *Water Environ. J.* **2006**, *20*, 43–47, doi:10.1111/j.1747-6593.2006.00030.x.
7. Wozniak, T. MBR design and operation using MPE-technology (Membrane Performance Enhancer). *Desalination* **2010**, *250*, 723–728, doi:10.1016/j.desal.2008.11.030.
8. Yoon, S.; Collins, J.H. A novel flux enhancing method for membrane bioreactor (MBR) process using polymer. *Desalination* **2006**, *191*, 52–61, doi:10.1016/j.desal.2005.04.124.
9. Munz, G.; Gori, R.; Mori, G.; Lubello, C. Powdered activated carbon and membrane bioreactors (MBRPAC) for tannery wastewater treatment: long term effect on biological and filtration process performances. *Desalination* **2007**, *207*, 349–360, doi:10.1016/j.desal.2006.08.010.
10. Remy, M.J.J. Low concentration of powdered activated carbon decreases fouling in membrane bioreactors, Wageningen University, the Netherlands, 2012.
